# Photo-generated carriers lose energy during extraction from polymer-fullerene solar cells

**DOI:** 10.1038/ncomms9778

**Published:** 2015-11-05

**Authors:** Armantas Melianas, Fabian Etzold, Tom J. Savenije, Frédéric Laquai, Olle Inganäs, Martijn Kemerink

**Affiliations:** 1 Biomolecular and Organic Electronics, Department of Physics, Chemistry and Biology, Linköping University, 58183 Linköping, Sweden; 2 Max Planck Research Group for Organic Optoelectronics, Max Planck Institute for Polymer Research, Ackermannweg 10, Mainz 55128, Germany; 3 Optoelectronic Materials Section, Department of Chemical Engineering, Delft University of Technology, 2628 BL Delft, The Netherlands; 4 Physical Sciences and Engineering Division, Material Science and Engineering, Solar and Photovoltaics Engineering Research Center, King Abdullah University of Science and Technology, Thuwal 23955-6900, Kingdom of Saudi Arabia; 5 Complex Materials and Devices, Department of Physics, Chemistry and Biology, Linköping University, 58183 Linköping, Sweden; 6 Department of Applied Physics, Eindhoven University of Technology, PO Box 513, 5600 MB Eindhoven, The Netherlands

## Abstract

In photovoltaic devices, the photo-generated charge carriers are typically assumed to be in thermal equilibrium with the lattice. In conventional materials, this assumption is experimentally justified as carrier thermalization completes before any significant carrier transport has occurred. Here, we demonstrate by unifying time-resolved optical and electrical experiments and Monte Carlo simulations over an exceptionally wide dynamic range that in the case of organic photovoltaic devices, this assumption is invalid. As the photo-generated carriers are transported to the electrodes, a substantial amount of their energy is lost by continuous thermalization in the disorder broadened density of states. Since thermalization occurs downward in energy, carrier motion is boosted by this process, leading to a time-dependent carrier mobility as confirmed by direct experiments. We identify the time and distance scales relevant for carrier extraction and show that the photo-generated carriers are extracted from the operating device before reaching thermal equilibrium.

## Abstract (web summary)

Organic photovoltaics rely on the collection of charge carriers generated in disordered materials. Here, the authors unify various experimental techniques and simulations to show that these devices operate out-of-equilibrium, questioning the typical assumption of quasi-equilibrium.

## Graphical abstract

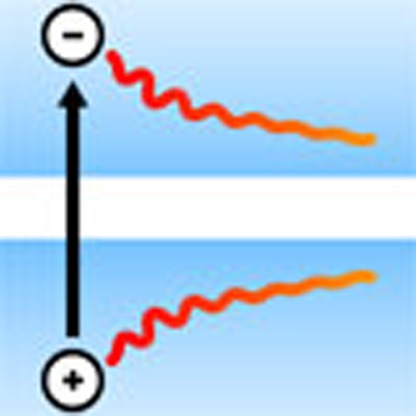

Organic solar cells, a carbon-based alternative to inorganic solar cells, can now perform at power conversion efficiencies over 10% (refs 1, 2). The photoactive layer in state-of-the-art organic photovoltaic (OPV) cells is most commonly a disordered mixture of a polymer donor and a fullerene-based acceptor. The mixture of the two, a so-called bulk-heterojunction, turns out to be a necessity for efficient exciton dissociation and free charge carrier generation. Following charge generation, the free charge carriers, as in inorganic solar cells, must be transported to the electrodes before they can deliver useful work in an external circuit. The energy of the extracted charge carrier population is critical as it is related by thermodynamics^3^ to the maximum attainable open-circuit voltage *V*_OC_, and thus to the power conversion efficiency of the photovoltaic device.

In analogy to modern, high-performance inorganic solar cells, for OPV cells it is often tacitly assumed that following photon absorption and free charge carrier generation—but before any transport to the electrodes occurs—the free charge carrier populations have already fully thermalized in their respective density of states (DOS). Thus, the free charge carriers are assumed to be transported at their corresponding equilibrium energy levels—lowest unoccupied molecular orbital (LUMO) for electrons and highest occupied molecular orbital (HOMO) for holes—in analogy to the conduction and valence bands of inorganic semiconductors. This assumption implies that no further charge carrier energy losses to thermalization can occur. In other words, a quasi-equilibrium situation is established and quasi-equilibrium Fermi-Dirac statistics can be employed. In silicon, for example, the photo-generated carriers thermalize by phonon scattering to their respective band edges in approximately 350 fs (ref. 4), making the quasi-equilibrium assumption valid. Despite lack of experimental proof this is often assumed to be also the case in OPV devices.

It is, in fact, well known that thermalization is especially important to consider in any semiconductor exhibiting strong energetic disorder, extending the DOS far into the bandgap^5^,^6^,^7^,^8^. For organic semiconductors, the DOS shape is commonly assumed to be Gaussian with a typical width *σ* around 0.1 eV. This leads to equilibrium energies^9^ of the order of *σ*^2^/*kT*≈0.4 eV away from the centres of the relevant levels: LUMO or HOMO. Hence, thermalization could potentially lead to a total energy loss (of both the electrons and the holes) as high as approximately 0.8 eV, an enormous number for a photovoltaic device. However, such large losses are in conflict with well-established empirical rules, which nevertheless indicate approximately 0.5 eV loss of open-circuit voltage^10^ to largely ill-understood causes^11^,^12^. In view of the above, it is surprising that thermalization in OPV devices has not yet been thoroughly investigated and that implicit assumptions of local quasi-equilibrium are widespread in the OPV community.

Here, we study thermalization over an exceptionally wide dynamic range. Our treatment includes several experimental techniques spanning up to 8 orders of magnitude in time. The results that follow are explained by a coherent quantitative model. Although in literature these techniques are mostly discussed in isolation, making it hard if not impossible to get the full picture, here all relevant time scales are investigated: a unification of the most commonly employed optical, electrical and numerical techniques to study OPV devices is presented. Our results indicate that in organic solar cells, the photo-generated carriers do not reach thermal equilibrium during their presence in the device. The mechanism of thermalization is in fact fundamentally different to that encountered in inorganic solar cells (excluding certain amorphous materials). At early times (1–100 ns) a substantial amount of energy of approximately 1–2*σ* is lost by fast diffusion-dominated carrier motion, followed by a slower loss of approximately 0.5–1*σ* during drift-dominated carrier extraction. Therefore, device models based on the assumption of quasi-equilibrium have to be reconsidered.

## Results

### Materials

We have studied photovoltaic blends of TQ1:PC_71_BM^13^,^14^,^15^,^16^,^17^,^18^,^19^ and PCDTBT:PC_61_BM^20^,^21^,^22^,^23^,^24^, yielding similarly high-power conversion efficiencies of 7%, and comparably high internal-quantum-efficiency values of approximately 90%. Full compound names are given in the Methods section. Both blends are known to be rather amorphous^14^,^22^. Nevertheless, the results that follow are expected to be general and not only apply to a broad range of disordered polymers but also to those of more semi-crystalline nature as will be further explained.

### Experiments define simulation parameters

To get a consistent physical picture at all relevant time scales, we have employed several transient experimental techniques that monitor charge carrier dynamics in OPV devices. The thermalization of photo-generated charge carrier populations (of positive polarons) was monitored by following the bleach signal of the time-resolved photo-induced absorption spectroscopy (TA)^23^,^24^ between 10^−13^ and 10^−6^ s. The transient mobility of the photo-generated carriers was monitored by time-resolved terahertz spectroscopy (THz)^16^, time-resolved microwave conductivity (TRMC)^17^,^25^ in combination with TA^18^ and time-delayed charge extraction experiments (pCELIV)^26^.

Experiments are complemented with kinetic Monte Carlo (MC) simulations based on the well-established Gaussian disorder model^8^, which accounts for the hopping carrier motion in the disorder broadened DOS and is particularly suited for this type of problem. In brief, the simulations account for: site occupation/state-filling effects; all Coulomb interactions, including image charges in metal electrodes (when present); electron-hole recombination and exciton diffusion. Whenever possible we used simulation parameters as obtained from experiments or fitting the model to experiments. Importantly, a single parameter set was used to simulate all experiments. To keep the number of simulation parameters to a minimum, the DOS of both the polymer and the PCBM were chosen to be single Gaussians. The combined rich experimental data set presented in this article and collected from previous work^19^,^24^,^27^ defined parameters for the kinetic MC model with little room for parameter adjustment. For further information regarding the model and which experiments defined the simulation parameters, see Supplementary Note 1, Supplementary Tables 1 and 2 and refs 19, 24, 27.

Finally, it is important to point out that we refer to ‘thermalization' or ‘relaxation' by hopping motion between localized sites. On-site thermalization/localization is expected to be very fast^28^ and thus unimportant for the time scales investigated here.

### Hole thermalization

**Figure 1:**
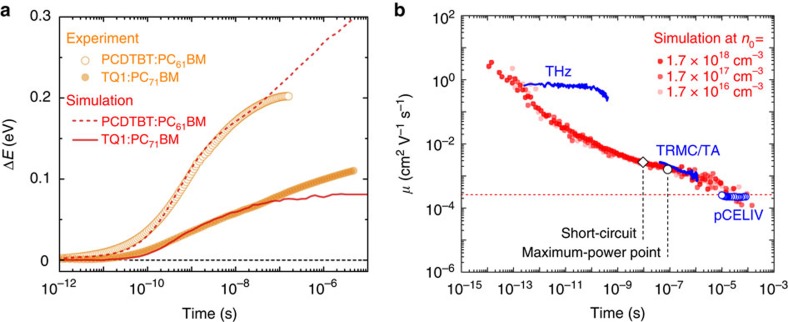
Hole thermalization dynamics and the time-dependent mobility. (**a**) Smoothed experimental data of the time-resolved bleach-peak shift in energy Δ*E* for TQ1:PC_71_BM (filled orange circles) and PCDTBT:PC_61_BM (empty orange circles) and the corresponding Monte Carlo simulations for TQ1:PC_71_BM (solid red line) and PCDTBT:PC_61_BM (dashed red line). The black dashed line indicates the centre of the hole DOS—the position of the HOMO level. (**b**) Time-dependent mean mobility of TQ1:PC_71_BM in THz and TRMC/TA experiments (blue lines), pCELIV experiment (blue open circles) and simulations at the indicated initial carrier densities *n*_0_ (red symbols). The red dashed line indicates the predicted mean equilibrium mobility as calculated from the simulation parameters^34^. Extraction times at short-circuit (black empty diamond) and at maximum-power point (black empty circle) mark the time scales relevant for electron extraction. THz results taken from ref. 16.

Following photon absorption, ultrafast exciton dissociation—the formation of charge-transfer states and free charge carriers—occurs on a time scale of hundreds of femtoseconds or less (see ref. 23 and Supplementary Fig. 1 for details), following which charge carrier thermalization commences. Figure 1a shows the thermalization of positive polarons (holes) as measured by the time-resolved photo-induced absorption spectroscopy under pulsed 532-nm laser illumination. A white-light-probe was used and the spectral shift of the bleach-signal-maximum Δ*E* was assigned to the thermalization of holes in the disorder broadened DOS^23^,^24^. See Supplementary Note 2 for why the photo-induced bleach (PB) band was chosen.

It has been argued that on the nanosecond timescale charge-transfer states can recombine into triplets on the polymer^29^, hence, the presence of triplets might limit the observable free hole energy loss to thermalization. On basis of general statistical considerations, two-particle (triplet) relaxation must be slower than single particle (free hole) relaxation: the number of final states at lower total energy available to the electron–hole pair, which is a joint DOS, is much (approximately quadratically) lower than for a single charge. As relaxation rates depend on the number of available final states, triplet relaxation must be significantly slower than free charge relaxation. Hence, the larger the ratio of holes in triplets to free holes, the smaller and slower is the hole thermalization observable via the PB. As the rate of triplet formation is pump-fluence dependent^29^,^30^,^31^, its limiting effect on the PB shift must also be dependent on pump-fluence. The absence of a pump-fluence dependence on the PB shift, see Supplementary Fig. 2 and refs 23, 24, therefore implies that the thermalization kinetics of Fig. 1a are not significantly affected by triplet formation and indicate free hole thermalization. For a more detailed discussion and the full 3D time-resolved and spectrally resolved data set of TQ1:PC_71_BM, see Supplementary Fig. 1 and Supplementary Note 2.

Hole thermalization energy losses are substantial: approximately 0.1 eV for TQ1:PC_71_BM (filled orange circles) and 0.2 eV for PCDTBT:PC_61_BM (empty orange circles). The approximately two times difference in the energy loss due to thermalization indicates that PCDTBT is considerably more energetically disordered than TQ1. This is also visible in the transient photoluminescence (TRPL) spectral peak shifts of pristine polymer films (Supplementary Fig. 3), where one observes approximately two times larger transient red shift for the PCDTBT than for the TQ1. For both systems, a substantial and relatively slow charge carrier thermalization energy loss occurs.

We find that the thermalization energy loss in TQ1:PC_71_BM is pump-fluence independent for pump-fluences of up to approximately 3.5 × 10^13^ photons per cm^2^ per pulse (Supplementary Fig. 2), which corresponds to initial carrier densities in the OPV device of up to approximately 1 × 10^18^ cm^−3^. Thermalization of holes in PCDTBT:PC_61_BM is also independent of light intensity^24^ for pump-fluences up to approximately 1 × 10^14^ photons per cm^2^ per pulse. This means that concentration (state-filling) effects are unimportant for the hole thermalization dynamics, a direct consequence of the system being out-of-equilibrium; whereas at quasi-equilibrium such concentrations do lead to strong state-filling effects^32^,^33^. Hence, the thermalization energy losses shown in Fig. 1a also occur (and in the same way) in devices operating at continuous 1 Sun illumination conditions^19^, where charge carrier densities are at least an order of magnitude smaller.

Figure 1a shows our MC model to successfully reproduce the hole thermalization dynamics for both TQ1:PC_71_BM (red solid line) and PCDTBT:PC_61_BM (red dashed line) up to a time scale of ≈100 ns. Simulations confirm that the thermalization of holes is concentration (pump-fluence) independent up to fractional DOS occupancies of the order of 0.01 (Supplementary Fig. 4). The mismatch at longer time scales is likely due to the fact that the real DOS of the material need not be single-Gaussian-shaped. The deviation suggests that below approximately 2*σ* the DOS tail decays more (TQ1:PC_71_BM) or less (PCDTBT:PC_61_BM) than prescribed by a Gaussian-shaped DOS. We note that an exponential DOS fails to reproduce the experimentally measured thermalization of holes in these blend systems.

Using a Gaussian-shaped DOS, Fig. 1a is essentially a two parameter fit: the energetic disorder *σ* of the HOMO defines the magnitude of the hole thermalization loss and the hole attempt-to-hop frequency *ν*_0_ defines the onset of thermalization. As suggested by experiments in Fig. 1a, PCDTBT should be considerably more energetically disordered than TQ1. From simulations, we find the energetic disorder of the HOMO level for PCDTBT:PC_61_BM blend to be 110 meV, which is in the range of the typically observed disorder values for OPV donor materials^27^,^33^,^34^. It is more than twice the remarkably low disorder *σ*≈45 meV found for the TQ1:PC_71_BM blend HOMO. A simple analysis of temperature-dependent space-charge limited current (SCLC) experiments in TQ1:PC_71_BM estimates *σ*≈52 meV (Supplementary Fig. 5), in good agreement with a low disorder. Such desirably low energetic disorder values are comparable to those reported for the semi-crystalline P3HT:PCBM blends *σ*≈40 meV (ref. 35). This is especially important as amorphous polymers are potentially easier to process. The reason for such a low energetic disorder of the HOMO level in TQ1:PC_71_BM is not entirely clear. However, as TQ1 is a liquid-crystalline polymer^14^, the low disorder is likely related to its rather rigid planar conformation of the conjugated backbone on a relevant distance scale. This would make the energies of the polymer segments, responsible for charge transport, insensitive to polymer backbone twists and turns in the otherwise disordered bulk-heterojunction mixture, thereby retaining only a small energy spread (energetic disorder) of the HOMO levels. A very similar argument was recently used to explain near-disorder-free charge transport in field-effect transistors^36^.

### Time-dependent mobility

As the hopping rate in a Gaussian DOS depends strongly on the energy of the hopping charge, it is expected that the mobility *μ* of the photo-generated charge carriers is time-dependent. In other words: as thermalization occurs downward in energy, carrier motion is boosted by this process. Transient mobility *μ*(*t*) has been investigated experimentally, however, only in: time scales longer than microseconds after photogeneration^26^, when most of the thermalization has already occurred, see Fig. 1a. As we will show below: mobilities measured at these time scales are not relevant for carrier extraction; early-time measurements over a limited time range^16^,^37^,^38^, whereas the full mobility transient between photogeneration and extraction should be considered; indirect experiments^39^ combined with simulations^19^,^24^,^40^; studies of pure polymers^41^,^42^ and PCBM^43^,^44^, results of which are not evidently comparable to OPV blends. Thus, to our knowledge, there is no conclusive experimental demonstration that mobility in operating OPV devices is time-dependent over all relevant time scales, and in fact it has been argued to be constant^37^,^45^,^46^. We now show by a combination of several direct experiments that charge carrier mobility in OPV devices is indeed time-dependent, and, importantly, is coupled to the energy loss outlined above.

Figure 1b shows the time-dependent mobility collected from various experiments (blue lines and symbols) and as predicted by our kinetic MC model for the indicated initial carrier densities *n*_0_ (red symbols) for TQ1:PC_71_BM. The model prediction is in excellent agreement with the experimental THz, TRMC in combination with TA (TRMC/TA) and pCELIV mobilities. See Supplementary Fig. 6 and Supplementary Note 3 for an explanation of how the experimental TRMC/TA mobility can be estimated. Although mobility in disordered organic semiconductors is known to be concentration dependent at quasi-equilibrium^32^,^33^, this is not necessarily the case when out-of-equilibrium; charge carriers reside at higher energies in the DOS where considerably more sites are accessible for transport, which significantly diminishes concentration effects. In fact, TRMC/TA experiments indicate that the time-dependent mobility is pump-fluence independent up to initial carrier densities of approximately 4 × 10^18^ cm^−3^ (Supplementary Fig. 6) and up to fractional DOS occupancies of 0.01 as confirmed by simulations in Fig. 1b and Supplementary Fig. 7. Therefore, for the relevant charge carrier densities encountered in operating OPV devices, the time-dependent mobility is concentration independent—state-filling effects are unimportant. Hence, the mobility of the photo-generated charges shown in Fig. 1b also decays (and in the same way) in devices operating at 1 Sun continuous illumination conditions^19^, for further details see Supplementary Figs 6–8 and Supplementary Note 3, where it is outlined how the TRMC/TA mobility can be estimated. As neither experimental technique can distinguish which charge carrier is observed, the experimental data in Fig. 1b are indicative of the mean charge carrier mobility. Therefore, also the mean simulated mobility is plotted. Supplementary Fig. 7 discusses the separate electron and hole contributions.

The partial mismatch between the THz experiment and the MC simulation can, to a large degree, be attributed to the fact that the calculated mobility is a (transient) frequency-independent (DC) mobility at a constant applied electric field, whereas the experimental mobility is frequency-dependent (AC) due to the time-varying electric field. When accounting for the time-varying electric field in the MC simulations, the THz mobility increases by about an order of magnitude, as expected on basis of earlier work^47^,^48^,^49^ and becomes less time-dependent, whereas the TRMC/TA mobility is unaffected, see Supplementary Fig. 9. Moreover, differences between fast intra-chain or intra-aggregate motion and slower inter-chain or inter-aggregate motion are not explicitly accounted for in the MC model, see ref. 50 for a complementary view. Intra-chain motion has previously been suggested to be predominantly probed in THz spectroscopy^38^,^49^. Full treatment of AC inter- and intra-chain motion is, however, beyond the scope of this work.

We do note that the pCELIV results indicate a final mobility decay at 10–20 μs (Supplementary Fig. 10) after which the charge carrier mobility for TQ1:PC_71_BM becomes constant. As the charge carrier mobility has reached its equilibrium value, also the charge carrier thermalization (of one of the charge carriers) must have been complete. Unfortunately, TA signals at such long time-delays are difficult to detect as few charge carriers survive recombination. It thus proved to be impossible to follow the hole thermalization in Fig. 1a up to 10 μs but the data do indicate at least a reduction in thermalization rate. The red dashed line indicates the mean equilibrium mobility calculated from the MC simulation parameters as suggested by Pasveer^34^. We find rather balanced electron *μ*_e_=3 × 10^−4^ cm^2^ V^−1^ s^−1^ and hole *μ*_h_=2 × 10^−4^ cm^2^ V^−1^ s^−1^ equilibrium mobilities, the mean of which *μ*=2.5 × 10^−4^ cm^2^ V^−1^ s^−1^ is in excellent agreement with the pCELIV experiment *μ*=2 × 10^−4^ cm^2^ V^−1^ s^−1^.

### Electron thermalization

So far we looked at hole thermalization dynamics only. In control TA experiments, we have also checked for electron signatures originating from fullerenes. However, both the PB and the photo-induced absorption of fullerenes were found to be very weak, making them hardly detectable in polymer:fullerene blends because of the presence of holes, the signatures of which are considerably stronger. Unfortunately, we are not aware of alternative experimental techniques that directly monitor the thermalization of the photo-generated electron population in PCBM. Hence, it is only indirectly, through the time-dependent mobility of Fig. 1b, that we know of electron thermalization. Nevertheless, the experiments presented in this article, those studied in previous work^19^,^24^,^27^ and especially Fig. 1b for TQ1:PC_71_BM, leave little room for simulation parameter adjustment. We are therefore confident to also make predictions for the electron thermalization dynamics on the basis of our kinetic MC model.

**Figure 2:**
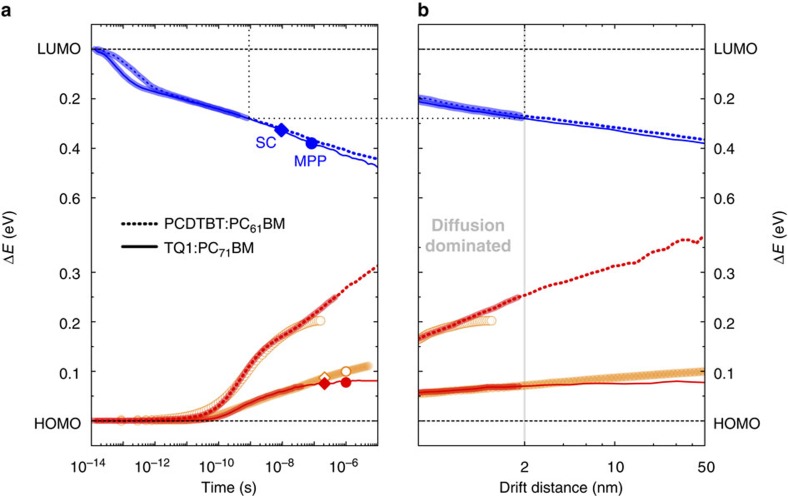
Thermalization of the photo-generated free charge carrier populations. (**a**) Thermalization dynamics of electrons (blue) and holes (red) with time for TQ1:PC_71_BM (solid lines) and for PCDTBT:PC_61_BM (dashed lines) as predicted by the model. Experimental data (orange symbols) are the same as in Fig. 1a. Symbols indicate the time at which the charge carrier has been extracted from the photovoltaic device at MPP (circles) and short-circuit conditions (diamonds). (**b**) Corresponding thermalization dynamics after the conversion of time to distance for an electric field strength of 0.2 V per 70 nm (MPP conditions). Thicker simulation traces in both panels indicate the distance region where motion is almost uniquely diffusive. The transition from diffusion- to drift-dominated carrier motion at a drift distance of ≈2 nm is indicated by the grey line. Black dotted lines indicate how the corresponding time and distance can be read from the figure.

Figure 2a shows the estimated free charge carrier thermalization losses for both blend systems studied in this work. Despite the large difference in hole thermalization dynamics (due to the difference in the energetic disorder of the HOMO levels), the thermalization dynamics of the electrons are found to be remarkably similar. This suggests that for efficient OPV devices and for fullerene loadings employed here, 2:5 for TQ1:PC_71_BM and 1:2 for PCDTBT:PC_61_BM, the energetic disorder of the PCBM is hardly affected by the choice of polymer and equals approximately 120 meV, the same value as reported for pristine PCBM^27^. This is further confirmed by identical time-dependent electron mobilities in both blends despite the slightly different electron hopping frequencies (Supplementary Fig. 11). This is highly unfortunate as a large disorder leads to large thermalization losses.

Although holes in PCDTBT:PC_61_BM and TQ1:PC_71_BM have similar hopping frequencies, as determined by their (similar) onsets of thermalization, the energetic disorder of the PCDTBT:PC_61_BM HOMO is considerably larger, as indicated by the larger losses to thermalization in Fig. 2. The larger energetic disorder therefore leads to a considerably lower time-dependent hole mobility for PCDTBT:PC_61_BM and thus an overall poor hole transport in this blend system^22^. Judging by thermalization losses alone, PCDTBT is inferior to TQ1 as a polymer donor.

### Thermalization mechanism

More insight can be extracted from the experimental and simulation data presented so far by transforming them to the amount of energy lost versus distance. This is accomplished by calculating the drift distance *d*(*t*) of the photo-generated charge carriers as


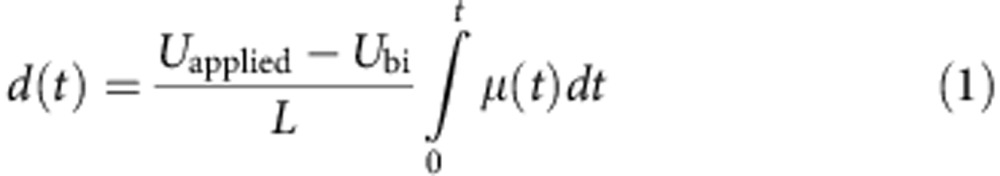


where *U*_applied_ is the applied voltage, *L* is the thickness of the photoactive layer, *μ*(*t*) is the time-dependent mean mobility of the charge carriers and *U*_bi_ is the built-in voltage of the photovoltaic device, which for well-performing OPV devices is typically around 1 V. As the simulation parameters responsible for the time-dependent mobility are all fixed by independent experiments, we can convert the thermalization time scales of TQ1:PC_71_BM and PCDTBT:PC_61_BM for both the electrons and the holes to mean drift distance. Moreover, as both the thermalization and, concomitantly, the time-dependent mobilities are pump-fluence independent, the conversion of time to distance is directly relevant to devices operating under 1 Sun continuous illumination conditions. Figure 2b shows the energy loss of the mean carrier versus drift distance from its location after the charge-transfer event, calculated for an electric field strength of 0.2 V per 70 nm—the maximum-power point (MPP) of the TQ1:PC_71_BM photovoltaic device (Supplementary Fig. 12). If ultra-fast long-range electron transfer^51^ took place, Fig. 2b would remain unchanged, as we are referring to energy losses from the location after the charge-transfer event and Δ*E*=0 eV for coherent electron transfer. For the associated time versus distance plots, see Supplementary Fig. 13.

Figure 2b indicates that charge carriers have lost a significant amount of excess energy of approximately 1–2*σ* before a net drift motion of a single lattice site (1.8 nm for TQ1:PC_71_BM) has occurred. This allows us to identify two time/distance scales relevant to charge carrier thermalization, separated by a drift distance of approximately 2 nm.

The large energy loss for a drift distance of less than 2 nm in Fig. 2b indicates that despite the absence of a net drift motion, a significant amount of energy is already lost. We now investigate why that is within the framework of our model, in which the photo-generated charge carriers can only lose energy by hopping to neighbouring sites. In fact, at the time the net drift distance of one lattice constant is reached (1–100 ns), significant hopping has already taken place: the average electron and hole in TQ1:PC_71_BM have performed approximately 7,000 and approximately 80 hops, respectively, and as many as 360 hops for the holes in PCDTBT:PC_61_BM. These surprisingly high numbers arise because most of the hops occur downward in energy, see Supplementary Note 4 for a more detailed explanation. This early-time motion is therefore almost entirely diffusive in nature, which is extremely unfortunate as the associated fast motion is not only wasted as heat but also does not directly contribute to charge extraction; indirectly, however, early-time fast motion does contribute to charge separation as was argued before^24^,^27^,^52^.

Following this large diffusive loss of excess energy, the drift component of motion becomes relevant. During the electric-field-directed motion to the contacts, the remaining excess energy is continuously, but not entirely, lost to further thermalization of approximately 0.5–1*σ*. At relevant electric field strengths, for example, at MPP and at short-circuit, charges are extracted from the photovoltaic device before reaching equilibrium. Compare for instance the actual electron relaxation of approximately 0.4 eV upon extraction (short-circuit and MPP symbols in Fig. 2a) to the electron equilibrium energy *σ*^2^/*kT*≈0.6 eV as predicted by the Gaussian disorder model. Device simulations at continuous 1 Sun illumination conditions further corroborate this observation^19^: the photo-generated charge carriers are extracted from the photovoltaic device before reaching quasi-equilibrium, see Supplementary Fig. 14 for a more detailed explanation.

### Relevant mobility experiments

Figure 1b illustrates that slow techniques such as pCELIV and SCLC are not very relevant for making meaningful statements on OPV device performance, as they probe the significantly lower mobility of the already equilibrated charges. The black symbols in Fig. 1b, which mark the extraction of electrons, indicate that the mean electron has been extracted from the photovoltaic device 2 orders of magnitude in time before the pCELIV experiment even begins. Note, moreover, that because of strong dispersion, approximately 50% of the carriers are extracted (much) earlier than the mean^19^,^53^. Therefore, the experimental TRMC/TA trace is more insightful and, most importantly, is the physically meaningful one. This is conclusively highlighted by the following fact: although both the electrons and the holes have nearly equal equilibrium mobilities in TQ1:PC_71_BM, their extraction times are orders of magnitude different, as can be inspected in Fig. 2a (symbols) and Supplementary Fig. 13. This is due to large differences in the time-dependent mobility, which via equation (1) are related to drift distance.

## Discussion

**Figure 3:**
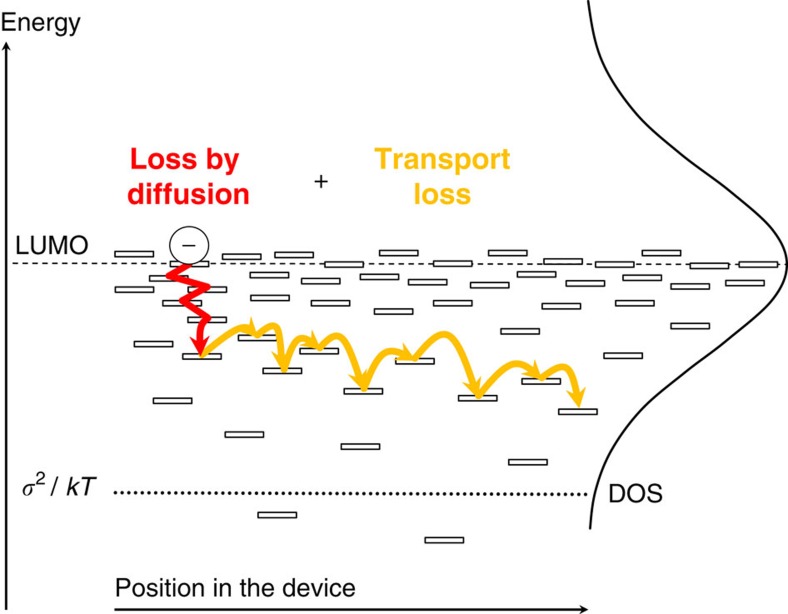
Schematic description of the free charge carrier thermalization. Charge carrier thermalization in OPV devices is a two-step process: first, most of the excess energy is lost by diffusion, as indicated by the red arrow going back-and-forth. At later time scales, the drift component of motion gradually becomes important and directed transport (yellow arrow) begins. During transport to the electrode, the remaining excess energy is continuously, but not entirely, lost by further thermalization. Charges are extracted from the photovoltaic device before reaching equilibrium at *σ*^2^/*kT*.

 The combined experimental and simulation data set forms a coherent picture that is schematically outlined in Fig. 3.

The mechanism of free charge carrier thermalization is fundamentally different from that encountered in modern, optimized inorganic solar cells, where band transport occurs and quasi-equilibrium Fermi-Dirac statistics can be used to describe the photo-generated charge carrier populations. The slow and incomplete thermalization in OPV devices invalidates the assumption of a quasi-equilibrium situation to be present in OPV devices. Concomitantly, the use of quasi-equilibrium Fermi-Dirac statistics to describe operating OPV devices is debatable at best.

An important message that Figs 1, 2, 3 above convey is that thermalization losses, especially during the diffusion-dominated early times, depend strongly on the material. They are therefore (partially) avoidable: for example, holes lose approximately 0.2 eV in PCDTBT:PC_61_BM and only 0.1 eV in TQ1:PC_71_BM. The thermalization of electrons in PCDTBT:PC_61_BM is rather similar to that in TQ1:PC_71_BM, approximately 0.3 eV. Although it has so far proven hard to find good alternatives to fullerene acceptors, the possibility to reduce electron thermalization loss, and hence gain in *V*_OC_, could be another motivation to continue this pursuit.

The energy losses presented in Figs 1 and 2, although not directly comparable to *V*_OC_ losses, are part of the origin of the commonly observed approximately 0.5 eV loss of the open-circuit voltage from the charge-transfer state^10^. For direct comparison, (i) changes in the free energy, which account for additional energy losses to entropy creation, and (ii) the influence of electrodes must be taken into consideration, which is outside the scope of this work.

Although the underlying mechanism of thermalization presented in this article is expected to be general, it must be pointed out that the magnitude of the thermalization loss (related to the DOS of the material) is also dependent on the active layer morphology; as supported by a recent study^2^, where a reduction of tail states in the DOS of PC_71_BM (due to PC_71_BM aggregation) has led to a gain in *V*_OC_. Crystalline polymer regions might further reduce thermalization losses via a smaller energetic disorder and enhanced charge carrier extraction. However, ordered regions are also lower in energy and can thereby even reduce the open-circuit voltage. Future research efforts should thus be focused to further minimize thermalization losses, which is possible by rational material design and active layer morphology control.

## Methods

### Full investigated material names

TQ1:PC_71_BM, poly[2,3-bis-(3-octyloxyphenyl)quinoxaline-5,8-diyl-alt-thiophene-2,5-diyl] (TQ1):[6,6]-phenyl-C_71_-butyric acid methyl ester (PC_71_BM). PCDTBT:PC_61_BM, poly[N-11''-henicosanyl-2,7-carbazole-alt-5,5-(4′,7′-di-2-thienyl-2′,1′,3′-benzothiadiazole)] (PCDTBT):[6,6]-phenyl-C_61_-butyric acid methyl ester (PC_61_BM).

### Investigated samples

PCDTBT:PC_61_BM samples were prepared at identical conditions as for the solar cell device^23^. For TQ1:PC_71_BM samples, a monolayer of poly(3,3′-([(9′,9′-dioctyl-9H,9′H-[2,2′-bifluorene]-9,9-diyl)bis(4,1-phenylene)]bis(oxy))bis(N,N-dimethylpropan-1-amine)) (PFPA-1) interface material was used to ensure the same active layer morphology as in the solar cell device. It was deposited on top of the used substrate before active layer deposition: Substrate/PFPA-1/TQ1:PC_71_BM. The active layer was then spin coated, using the same settings as for the solar cell device: Glass/ITO/PFPA-1/TQ1:PC_71_BM/LiF/Al. See Supplementary Fig. 12 for device characteristics. Therefore, in TA, TRMC/TA and TRPL experiments, samples with the following structure were investigated: Substrate/PFPA-1/TQ1:PC_71_BM. Only in THz experiments, Glass/TQ1:PC_71_BM samples were measured. In pCELIV experiments, samples with an inverted device architecture Glass/Al/TiOx/PFPA-1/TQ1:PC_71_BM/PEDOT:PSS were used instead. This turned out to be a better device structure for this type of measurement, where it is necessary to minimize the leakage of the photo-generated charge carriers out of the device during the μs delay time. For SCLC measurements, ITO/PEDOT:PSS/TQ1:PC_71_BM/PEDOT:PSS (PH1000) hole-only devices were investigated.

### Time-resolved optical/electro-optical experiments

In all experiments, samples were pumped at 532 nm at similar pump-fluences. Specific pump-fluence values used in experiments can be found in the Supplementary Information. THz experiments are described in ref. 16. TRMC experiments were performed as described in refs 17, 25. For an explanation of how the TRMC/TA mobility was estimated see Supplementary Fig. 6 and Supplementary Note 3. pCELIV experiments are described in refs 17, 19.

Transient absorption experiments were carried out with a home-built pump-probe setup. The output of a titanium:sapphire amplifier (Coherent LIBRA HE, 3.5 mJ, 1 kHz, 100 fs) was used to seed two independent optical parametric amplifiers (Coherent OPerA Solo), of which one was used to generate a pump pulse. The second OPA was used generate a seed pulse for supercontinuum generation, which served as the broadband probe pulse. For supercontinuum generation in the range of 500–1,100 nm, a 1,300-nm pulse was focused into a 3-mm c-cut sapphire crystal. Time resolution was obtained by delaying the pump pulses on a motorized delay stage between 150 fs and 4 ns with respect to the probe pulse. For experiments in the time range from 1 ns to 1 ms, the excitation pulse was provided by an actively Q-switched Nd:YVO_4_ laser (AOT Ltd. MOPA) at 532 nm. The delay between pump and probe was controlled by an electronic delay generator (Stanford Research Systems, DG535). TA measurements were performed at room temperature under a dynamic vacuum of <10^−5^ mbar. The transmission spectrum of the probe pulses was measured with a linear silicon photodiode array and successive probe pulses were used to determine the change in transmission induced by the pump pulse. Data collection and analysis were performed with home-built readout electronics and a LabView-based data acquisition and analysis software.

### MC simulations

For a detailed description of the MC model and which experiments defined the simulation parameters, see Supplementary Note 1 and Supplementary Tables 1 and 2.

## Additional information

**How to cite this article:** Melianas, A. *et al.* Photo-generated carriers lose energy during extraction from polymer-fullerene solar cells. *Nat. Commun.* 6:8778 doi: 10.1038/ncomms9778 (2015).

## Supplementary Material

Supplementary Information Supplementary Figures 1-14, Supplementary Tables 1-2, Supplementary Notes 1-4 and Supplementary References.
