# Supplementary material for: Photo-generated carriers lose energy during extraction from polymer-fullerene solar cells
Source: Nat Commun. 2015 Nov 5;6:8778. doi: 10.1038/ncomms9778 (PMC4659933; doi:10.1038/ncomms9778)
Supplement: Supplementary Information — Supplementary Figures 1-14, Supplementary Tables 1-2, Supplementary Notes 1-4 and Supplementary References. [file ncomms9778-s1.pdf]

Supplementary Figures

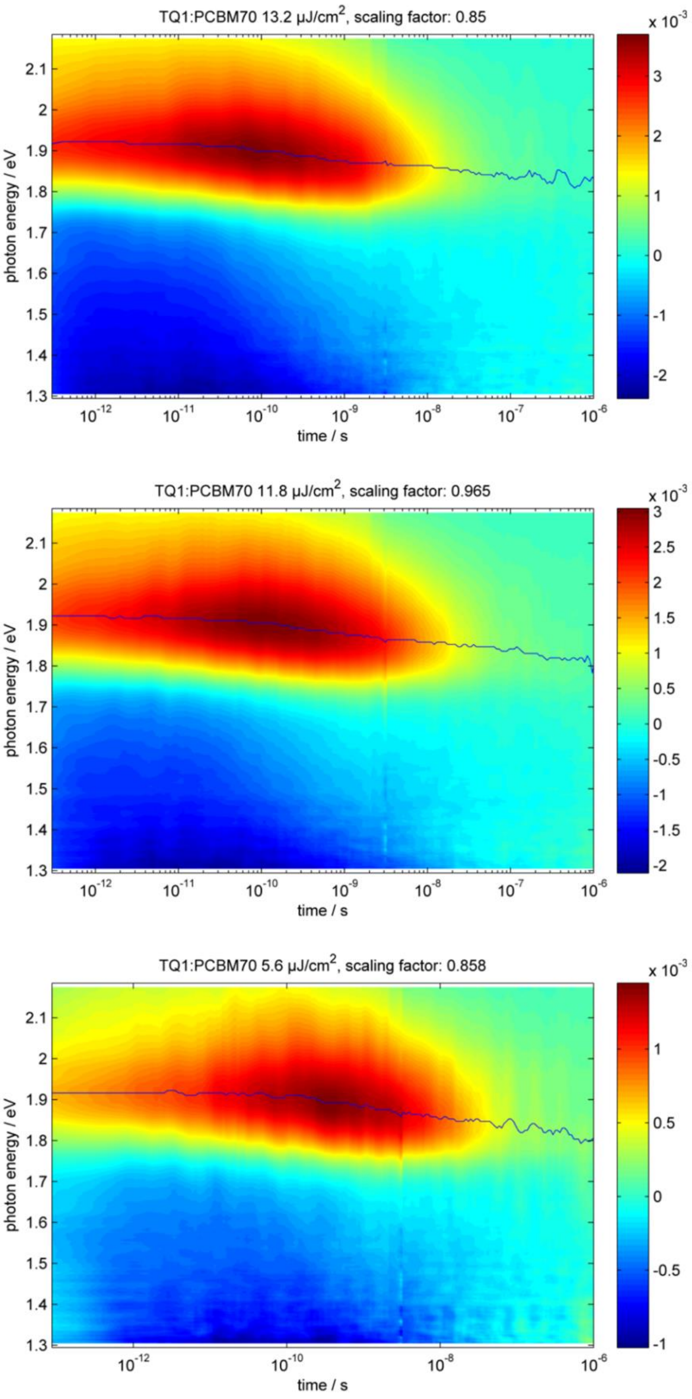

Supplementary Figure 1 | TA data surfaces of TQ1:$PC_{71}BM$ for the indicated pump-fluences. Solid lines indicate the bleach-signal-maximum, which is assigned to the thermalization of the holes in the disorder broadened density of states[1,2], see Supplementary Fig. 2. The formation of charge-transfer states and free charge carriers in TQ1:$PC_{71}BM$ occurs on a time scale of hundreds of fs or less as can be inspected from the photo-induced absorption band. The barely visible boundary line at ≈3 ns is due to a change from a mechanically to an electronically delayed excitation pulse, scaling factors are indicated in the figure.

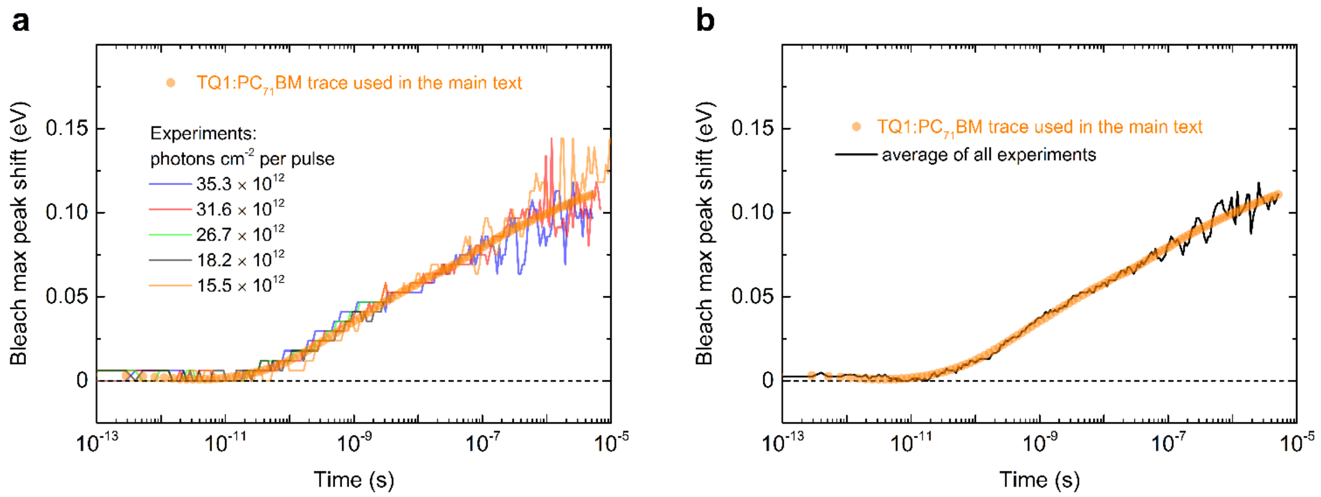

Supplementary Figure 2 | Hole thermalization dynamics in experiment. (**a**) Experimental bleach-peak-maximum spectral shifts with time for the indicated pump-fluences. Spectral shifts are pump-fluence independent within the accuracy of our experimental setup. We observe no significant exciton diffusion, which would lead to an additional red-shift of the bleach peak prior to exciton dissociation, fully consistent with Supplementary Fig. 1, indicating ultrafast exciton dissociation on the timescale of hundreds of fs or less. The traces were first averaged and then smoothed, orange symbols indicate the result, which was used in the main text. (**b**) Comparison of the average of all pump-fluences to the smoothed curve as used in the main text and shown in panel (a).

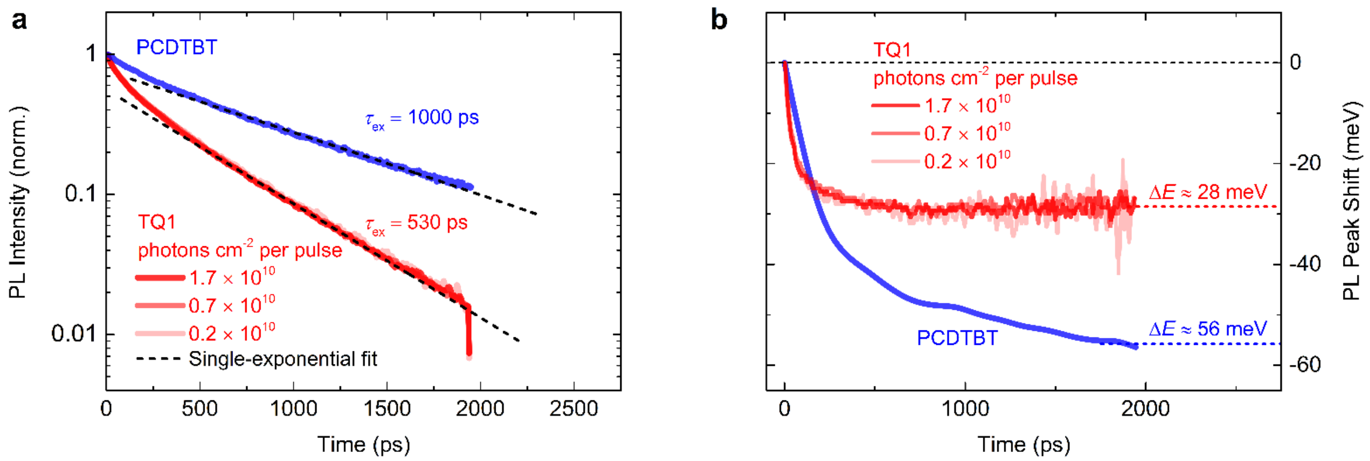

Supplementary Figure 3 | Low energetic disorder of TQ1 indicated by transient photoluminescence which was measured by a STREAK camera. (**a**) Shows the transient photoluminescence (TRPL) intensity decay of pristine TQ1 (red) and PCDTBT (blue) polymer films, from which the exciton lifetime τ_{ex} was estimated for MC simulations of the blends. The slower decay component was fitted by a single-exponential. Samples were excited at 400 nm. (**b**) Shows the corresponding TRPL peak shifts. The notably smaller PL peak shift in the case of TQ1 (red) as compared to PCDTBT (blue) indicates a considerably smaller energetic disorder in TQ1. Dashed lines are a guide to the eye. Results for PCDTBT taken from ref. 2.

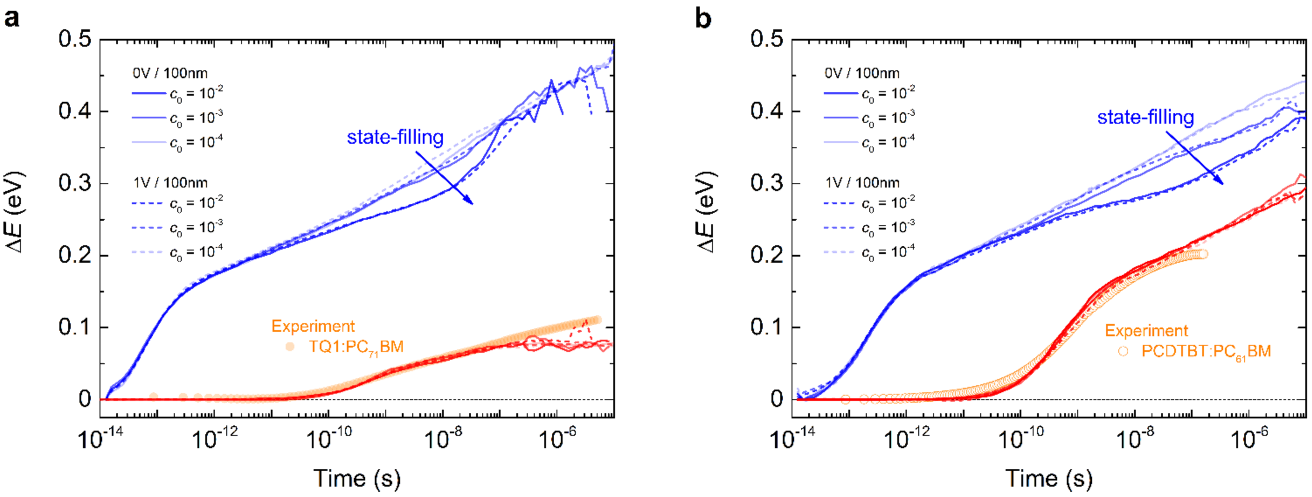

Supplementary Figure 4 | Hole and electron thermalization dynamics in simulation. Simulated hole (red) and electron (blue) thermalization dynamics in time for the indicated fractional occupations c_0 of the density of states and at two different fields of 0V/100nm and 1V/100nm (**a**) TQ1:PC_{71}BM (**b**) PCDTBT:PC_{61}BM. Thermalization is found to be independent of both fractional occupation (pump-fluence) and field, except at longer times for electron concentrations exceeding $c_0 \approx 10^{-3}$, where state-filling effects are observed. Nevertheless this is not so important as a) it does not affect the time-dependent mobility of electrons, see Supplementary Fig. 7 b) only the lowest fractional occupations are relevant for OPV devices operating at 1 sun continuous illumination conditions. Therefore the lowest pump-fluence curves were used for the conversion of time to distance and for estimates of the energy loss versus distance.

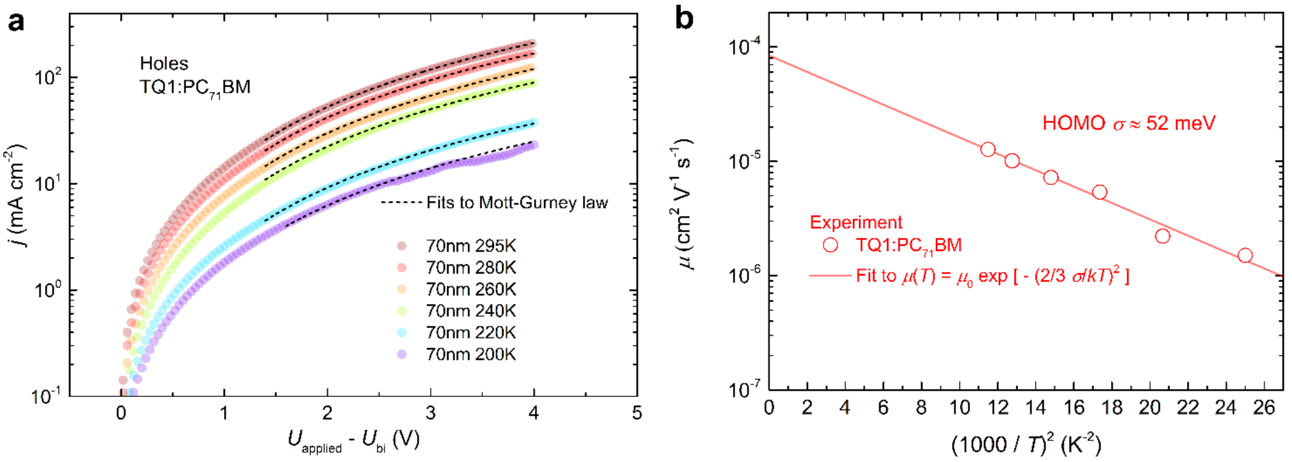

Supplementary Figure 5 | Low energetic disorder of TQ1 indicated by temperature-dependent SCLC. (**a**) TQ1:PC_{71}BM hole-only diode I-V curves in the dark at indicated temperatures. Results were obtained on active layers from another TQ1 batch that is nominally identical to the one in the main text. Dashed black lines are fits according to the Mott-Gurney law. Active layer thickness is the same as for the OPV device in the main text. (**b**) Estimated TQ1:PC_{71}BM HOMO level energetic disorder as predicted by the GDM[3].

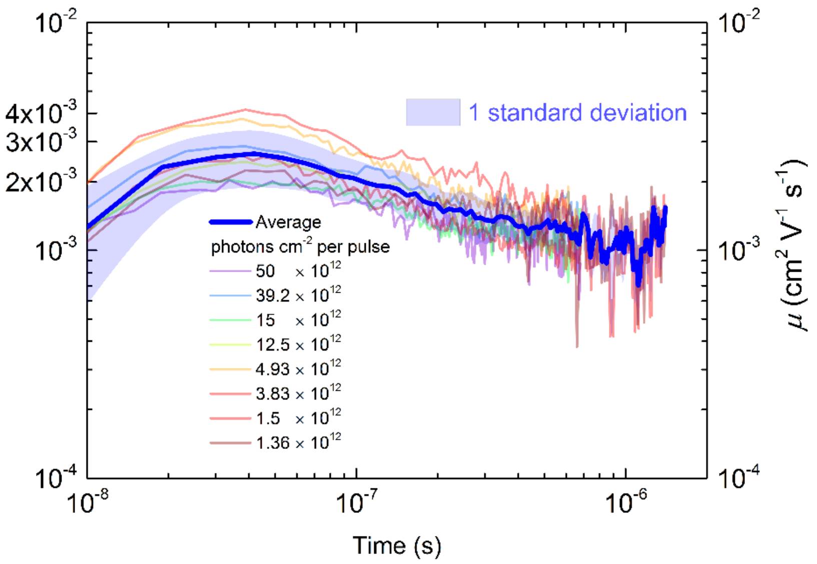

Supplementary Figure 6 | TRMC/TA mobility was estimated by dividing the TRMC results[4] $\sigma(t)$ by the PIA[5] estimate of $n(t)$ at the indicated PIA pump-fluences. Since thermalization of the charge carriers is found to be pump-fluence independent (Supplementary Fig. 2 and 4), and since charge carrier thermalization is coupled to their motion, also the time-dependent mobility $\mu(t)$ is expected to be pump-fluence independent. We find that this is indeed the case by estimating TRMC/TA mobility in the experimentally accessible pump-fluence range of ≈1-50 × 10^{12} photons cm^{-2} per pulse, which corresponds to charge carrier densities ≈1-40 × 10^{17} cm^{-3}. We do note that the TRMC conductivity pump-fluences were slightly different than those of PIA but no more than a factor of two, which is within the experimental error at which the pump-fluences can be determined. Nevertheless at the 8 experimentally accessible (by both techniques) pump-fluences the error in $\mu(t)$ is remarkably small – an identical result is obtained within a factor of two. Our Monte Carlo model reproduces this independence, as is indicated in Supplementary Fig. 7 and Figure 1b of the main text. The trace presented in the main text is averaged over all pump-fluences and has the non-physical data removed: (1) the noisy long-time data and (2) the initial TRMC signal rise, which is caused by the finite response time of the microwave cavity. Further details on how the TRMC/TA mobility was estimated are given in the Supplementary Note 3.

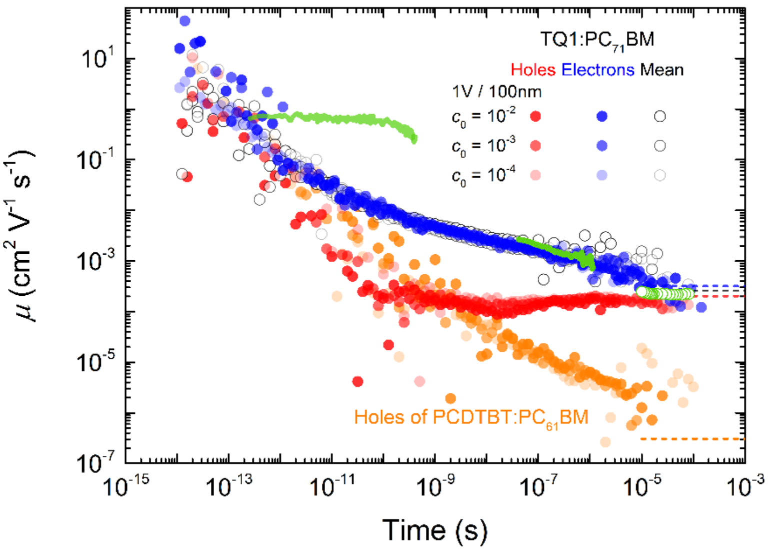

Supplementary Figure 7 | Comparison of simulated time-dependent mobilities at indicated fractional DOS occupancies c_0 to experiments (green). The time-dependent mobility of both the electrons and the holes is pump-fluence independent in agreement with TRMC/TA experiments. Electrons of PCDTBT:PC_{61}BM are not shown as their time-dependent mobility is identical to that of TQ1:PC_{71}BM, see Supplementary Fig. 11. Green lines and open green circles are experiments as shown in Figure 1b of the main text. The dashed lines indicate the equilibrium mobilities of TQ1:PC_{71}BM as estimated from the Monte Carlo parameters using the parametrization suggested by Pasveer[6]. For PCDTBT:PC_{61}BM our Monte Carlo parameters predict a very low hole equilibrium mobility of only $\mu_h = 3 \times 10^{-7}$ cm^2 V^{-1} s^{-1} as indicated by the orange dashed line. Only at the time scale of the TRMC/TA experiment the transient electron and hole mobilities differ significantly: the TRMC/TA mobility estimate (Figure 1b of the main text) should be mostly dominated by the larger electron mobility[4,7]. At shorter (THz) and longer (pCELIV) time scales the situation is expected to be more balanced.

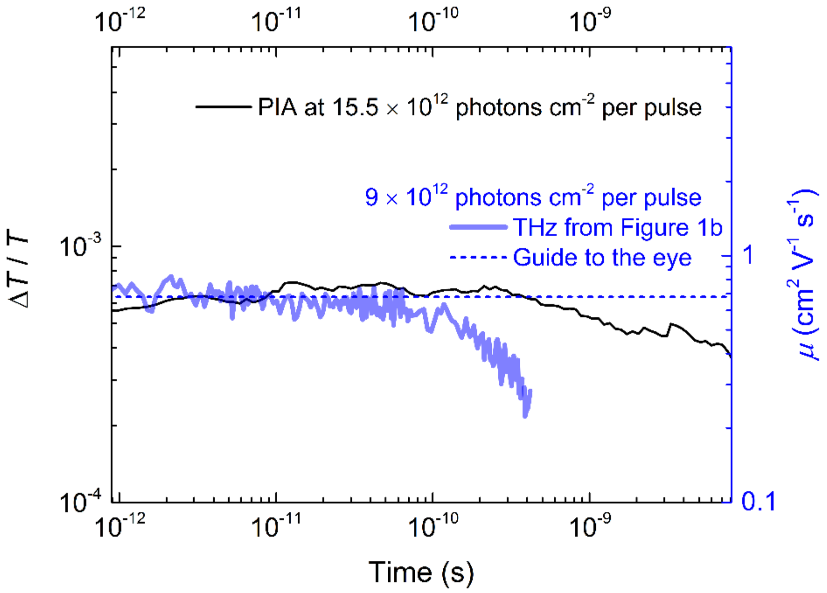

Supplementary Figure 8 | THz signal comparison to PIA. Charge carrier density $n(t)$ as measured by the PIA (solid black line) is time-independent on the time scale of the THz mobility decay (blue line), even at the almost twice as high pump-fluence used for the PIA as for the THz spectroscopy. For the PIA signal at the same pump-fluence, which what this trace should be compared to, see ref. 12. The dashed blue line is a guide to eye.

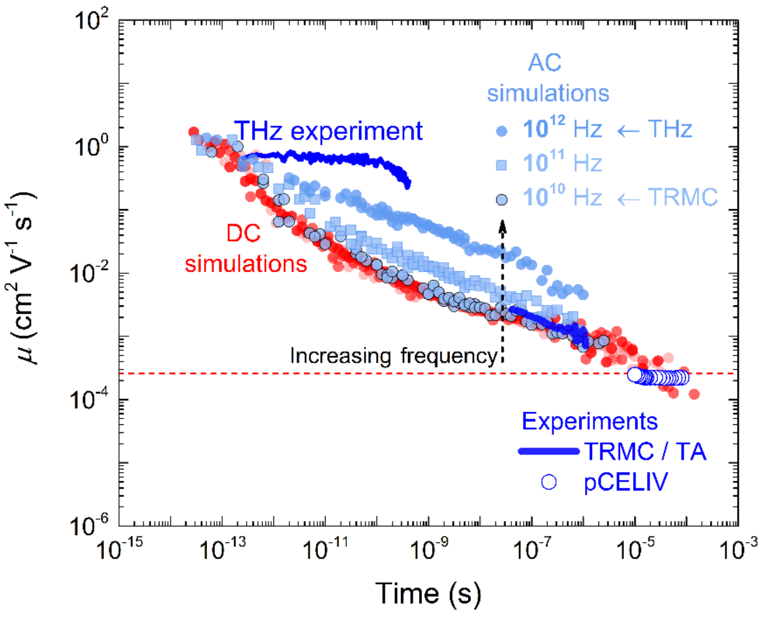

Supplementary Figure 9 | Simulated AC effects on the time-dependent mobility in TQ1:PC$_{71}$BM. Simulations are nominally identical to those described in the main text and Supplementary Fig. 7, except for the applied electric field, which is now harmonically modulated (AC) – a sinusoidal electric field $E(t)=E_0\sin(2\pi ft)$ at the indicated frequencies f was applied. The amplitude of the AC electric field, E_0, was chosen to match that encountered in THz experiments ≈10^5 V/m[8]. Simulated mean mobility is shown in the figure, mostly dominated by the higher electron mobility. Note, that at frequencies used in TRMC experiments ≈10^{10} Hz AC effects are unimportant, as the simulated mobility trace at 10^{10} Hz overlaps with the DC simulation and the TRMC/TA experiment. Mobility is higher at higher frequencies as shorter distances are probed[9–11]: at high frequencies carriers predominantly hop back-and-forth between sites of similar energy, and are thus less prone to encounter high energy barriers (difficult hops), as is the case for long-distance DC transport probed by TRMC/TA and pCELIV. However, full treatment of non-equilibrium AC conductivity is beyond the scope of this work.

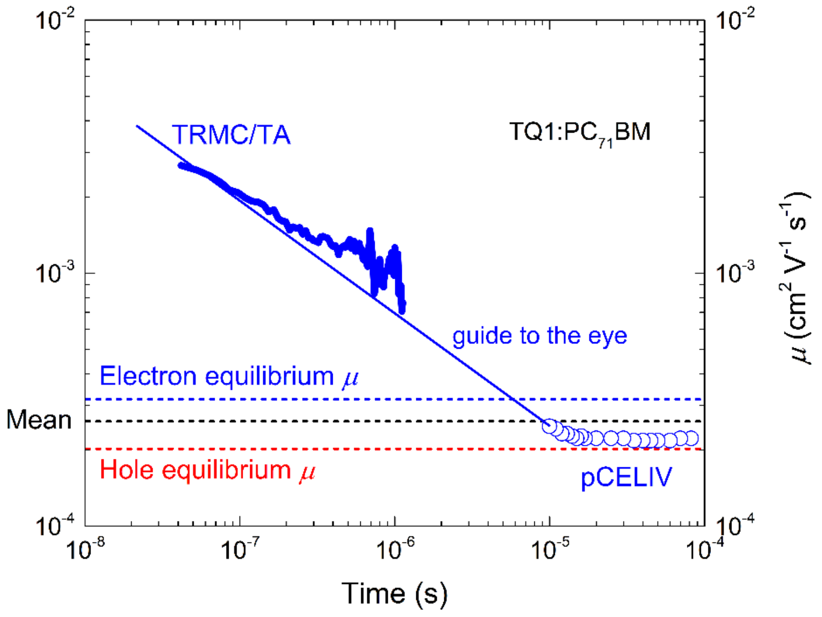

Supplementary Figure 10 | TRMC/TA and pCELIV mobilities zoomed-in. The earlier time 10-20 µs pCELIV results indicate a minor rise, which are very likely connected to the TRMC/TA mobilities. The dashed lines show the equilibrium electron and holes mobilites estimated from the Monte Carlo parameters using the parametrization suggested by Pasveer[6]. The dashed black line is the corresponding mean.

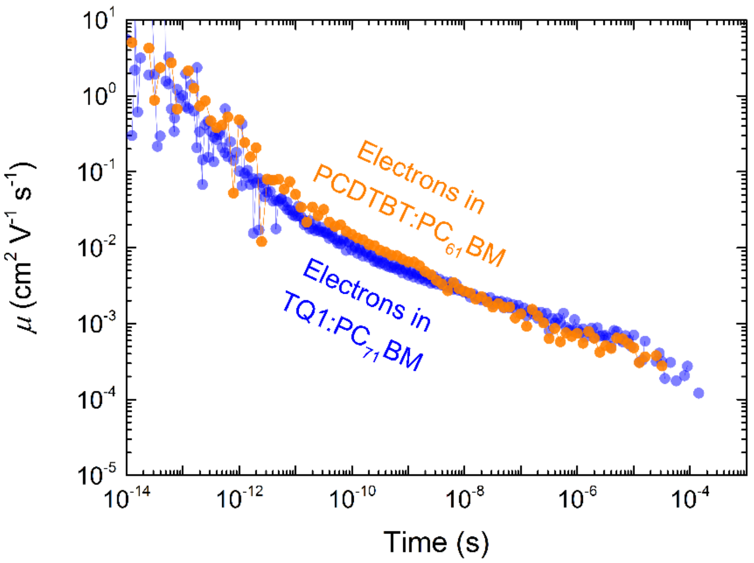

Supplementary Figure 11 | Time-dependent electron mobility in TQ1:$PC_{71}BM$ and PCDTBT:$PC_{61}BM$ are found to be close to identical, despite the factor of ≈3 smaller electron hopping frequency in PCDTBT:$PC_{61}BM$, see Supplementary Table 1, 2. This difference is attributed to both the uncertainty in the parameter fitting procedure and the different structure and symmetry of the C_{60} and C_{70} cages.

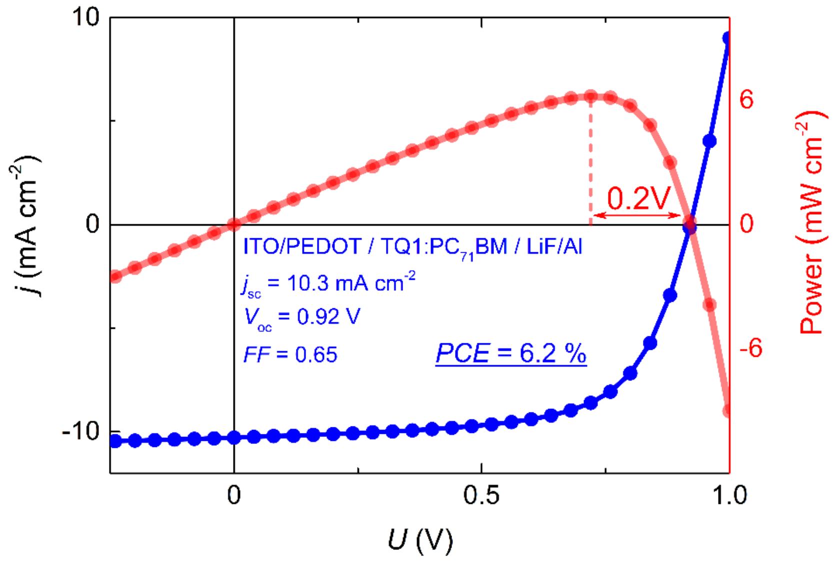

Supplementary Figure 12 | Photovoltaic performance of TQ1:$PC_{71}BM$ diodes. Devices used for measurements in the main text perform at 6.2% in standard geometry and have an IQE≈90 %[13]. Device characteristics are indicated in the figure as is the maximum-power point (MPP) at an effective reverse bias of 0.2V. The highest power conversion efficiency reported for TQ1:$PC_{71}BM$ based photovoltaic diodes is 7%[14].

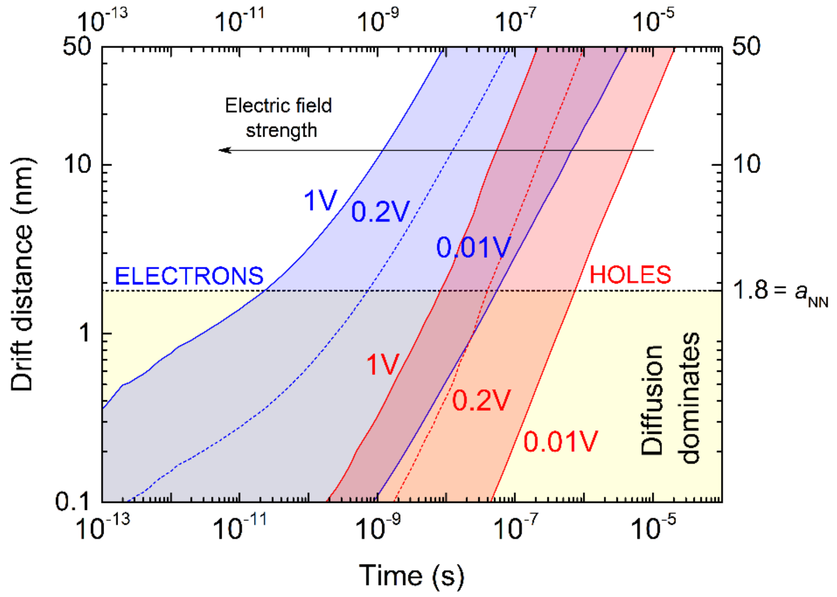

Supplementary Figure 13 | Drift distance versus time for the relevant bias points of the TQ1:PC_{71}BM I-V curve. Calculated for an active layer thickness of 70 nm. Solid lines (blue for electrons and red for the holes) show the drift distance with time for the indicated effective ($U_{\mathrm{applied}} - U_{\mathrm{bi}}$) reverse bias in the OPV device: 1V thus corresponds to short-circuit (SC) and 0.2V to maximum-power point (MPP) conditions. The black arrow indicates increasing electric field strength, at higher fields the traces are shifted to earlier times. Colored dashed lines indicate traces at MPP, used for Figure 2 of the main text. The shaded areas are a guide to the eye for the voltages not shown. The yellow shaded area indicates the diffusion-dominated region where drift motion has not yet accumulated to the hopping distance a_{NN} = 1.8 nm, indicated by the black dashed line. At early times the convergence of drift distance to the maximum transport distance, as implied by the Einstein-Smoluchowski relation, was ensured.

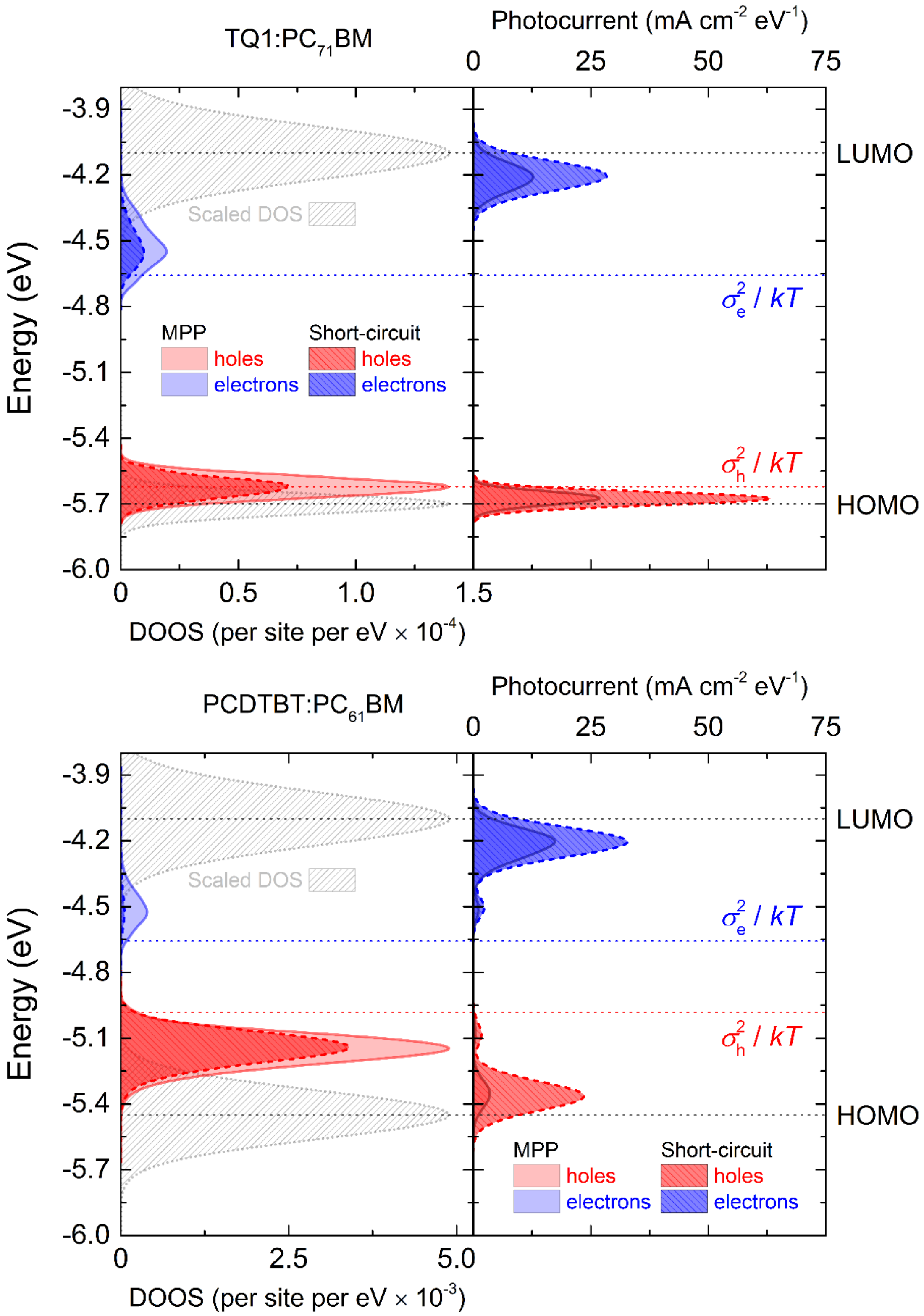

Supplementary Figure 14 | Simulated energy level-resolved density of occupied states (DOOS) and the corresponding photocurrent of TQ1:$PC_{71}BM$ (top) and PCDTBT:$PC_{61}BM$ (bottom) photovoltaic devices at continuous ≈1 sun illumination conditions. The energy-resolved DOOS (left plot) of holes (red) and electrons (blue) present in the device are shown by the color shaded regions at short-circuit (1V/70nm) and at MPP (0.2V/70nm) conditions as indicated in the legend. The corresponding energy level-resolved photocurrent is shown in the right plot. In both blends electrons have not

reached thermal equilibrium during their presence in the device, for example compare the electron DOOS to their estimated equilibrium energy σ^2/kT, indicated by the dotted blue line. Also the hole population in PCDTBT:PC_{61}BM has not reached its equilibrium energy σ^2/kT due to the relatively large disorder of the PCDTBT HOMO. Although the above figure indicates that in case of TQ1:PC_{71}BM the hole population has reached its equilibrium energy σ^2/kT (dotted red line), it must be noted that 1) The relatively slow transition to this state is not visible in the figure, however charge carriers still have to undergo thermalization to reach this state, Figure 1a of the main text shows this transition. 2) The lesser thermalized charge carriers generate most of the photocurrent via their considerably higher (time-dependent) mobility and are thus extracted before reaching thermal equilibrium, see the corresponding energy level-resolved photocurrent in the right plot. This is generally the case, for example compare for other populations. 3) The experiment of Figure 1a indicates further thermalization that is not captured in the model prediction, see Figure 1a.

Supplementary Tables

Supplementary Table 1 | Overview of TQ1:$PC_{71}BM$ parameters used in the simulations. Parameters in grey have no effect on the results but are shown for completeness. HOMO and LUMO refer to the orbitals of the effective medium.

Parameter	Value + unit	**Experiment**
a_{NN}	1.8 nm	Ref[6,15] and fit to transient photocurrent[16]
ΔE^{LUMO}	0.8 eV	Ref[17] and SW voltammetry[18]
E^{LUMO}	4.1 eV	Ref[17]
E^{HOMO}	5.7 eV	SW voltammetry[18]
σ^{LUMO}	0.120 eV	Fit to transient photocurrent[16], TRMC/TA and pCELIV
σ^{HOMO}	0.045 eV	Fit to hole thermalization and pCELIV
ν_0^{LUMO}	1×10^{13} s^{-1}	Fit to transient photocurrent[16], TRMC/TA and pCELIV
ν_0^{HOMO}	1×10^{9} s^{-1}	Fit to hole thermalization and pCELIV
ν_{CT}	1×10^{8} s^{-1}	Fit to transient photocurrent[16] and IQE≈85%[13]
ν_{ex}	1.9×10^{9} s^{-1}	TRPL in Supplementary Fig. 3
R_0/a_{NN}	2.2	Ref[17]

Supplementary Table 2 | Overview of PCDTBT:$PC_{61}BM$ parameters used in the simulations. Parameters in grey have no effect on the results but are shown for completeness. HOMO and LUMO refer to the orbitals of the effective medium.

Parameter	Value + unit	**Experiment**
a_{NN}	2.6 nm	Ref[6,15], fit to hole thermalization[2] and recombination dynamics[2]
ΔE^{LUMO}	0.5 eV	Ref[17,19]
E^{LUMO}	4.1 eV	Ref[17]
E^{HOMO}	5.45 eV	Ref[19]
σ^{LUMO}	0.120 eV	T dependent SCLC of pristine PCBM[17] and recombination dynamics[2]
σ^{HOMO}	0.110 eV	Fit to hole thermalization[2] and recombination dynamics[2]
ν_0^{LUMO}	3×10^{12} s^{-1}	T dependent SCLC of pristine PCBM[17] and recombination dynamics[2]
ν_0^{HOMO}	1×10^{9} s^{-1}	Fit to hole thermalization[2] and recombination dynamics[2]
ν_{CT}	5×10^{7} s^{-1}	Recombination dynamics[2]
ν_{ex}	1×10^{9} s^{-1}	TRPL[2]
R_0/a_{NN}	2.4	TRPL[2]

Supplementary Notes

Supplementary Note 1 | Model description. The kinetic Monte Carlo model is implemented on a simple cubic grid – each site is six-fold coordinated. The nearest neighbor hopping distance a_{NN} then equals the lattice constant and relates to the total DOS as $a_{\mathrm{NN}} = N_0^{-1/3}$. Supplementary Table 1, 2 give an overview of the parameters used to generate the simulations shown in this work; below the model and its parameters are explained in detail.

We use the Miller-Abrahams expression to quantify, with the least number of parameters, the nearest-neighbor hopping rate of a charge carrier from an initial state i with energy E_{i} to a final state f with energy E_{f} as

$$\nu_{\mathrm{if}} = \begin{cases} \nu_0 exp\left(-\dfrac{E_{\mathrm{f}} - E_{\mathrm{i}} \pm q\mathbf{r}_{\mathrm{if}} \cdot \mathbf{F} + \Delta E_{\mathrm{C}}}{k_{\mathrm{B}}T}\right), & \Delta E > 0 \\ \nu_0, & \Delta E \leq 0 \end{cases} \qquad (1)$$

Here $\mathbf{F}$ is the external electric field, $\mathbf{r}_{\mathrm{if}}$ the vector connecting initial and final sites, ν_0 the attempt-to-hop frequency, and q the positive elementary charge. The + (−) sign refers to electron (hole) hopping. The term ΔE_{C} is the change in Coulomb energy and is calculated by explicit evaluation of the interaction of the moving charge with (a) all other charges in the simulated device and (b) their image charges, as well as of the interaction of the image charges of the moving particle with (c) the particle itself and (d) all other particles. Image charges arise when metallic contacts are present; the number of image charges accounted for in the simulations is increased till the resulting effective Coulomb potential no longer changes. In order to avoid divergences at zero separation, the Coulomb interaction between a pair of (unlike) charges $E_{\mathrm{C}} = -q/4\pi\varepsilon_0\varepsilon_{\mathrm{r}}r_{\mathrm{eh}}$ with $\varepsilon_0\varepsilon_{\mathrm{r}}$ the dielectric constant (ε_{r} = 3.6) and r_{eh} the electron-hole distance is truncated at minus the approximate exciton binding energy of $E_{\mathrm{b}}^{\mathrm{ex}}$ = 0.5 eV. The single-particle site energies E_{i} are drawn from a Gaussian distribution function

$$f(E_{\mathrm{i}}) = \frac{1}{\sqrt{2\pi\sigma^2}} \exp\left(-\frac{(E_{\mathrm{i}} - E_0)^2}{2\sigma^2}\right) \qquad (2)$$

with E_0 the mean energy and σ the broadening of the total density of states (DOS) N_0. The HOMO and LUMO energy of a single site are assumed to be uncorrelated.

We define an effective hopping medium with (different) electron and hole hopping parameters ν_0 and σ that correspond to the donor HOMO and acceptor LUMO levels, respectively. The driving force for charge transfer is implemented via an on-site electron-hole repulsion with a magnitude that equals the LUMO level offset $\Delta E^{\mathrm{LUMO}} = E_{\mathrm{donor}}^{\mathrm{LUMO}} - E_{\mathrm{acceptor}}^{\mathrm{LUMO}}$ between donor and acceptor. More elaborate accounts of the bulk heterojunction double the number of unknown hopping parameters and force one to make statements about the hierarchical length scales in the phase separated morphology. More importantly, the transport and relaxation, in which we are mainly interested here, occurs in the levels that are explicitly accounted for in the present treatment.

A consequence of the neglect of the phase separated morphology is that the model would not be able to reproduce an eventual reduced bimolecular recombination rate due to electrons and holes being spatially separated in different phases. However, at the conditions investigated here 'non-reduced' bimolecular recombination is already unimportant.

Spatially direct excitons, formed by an electron and a hole on the same site, can recombine with rate ν_{ex}. Similarly, when sitting on neighboring sites they form a CT complex that can recombine with rate ν_{CT}. This implies that mono- and bimolecular recombination are treated on equal footing, as recombination rates of exciton and CT species do not depend on the history of the constituent charges. Exciton diffusion by the Förster resonant energy transfer (FRET) mechanism is explicitly accounted for. The transition rate is evaluated as

$$\nu_{\mathrm{if}}^{\mathrm{F}} = \nu_{\mathrm{ex}} \left(\frac{R_0}{r_{\mathrm{if}}}\right)^6 \Theta(E_{\mathrm{i}}^{\mathrm{ex}} - E_{\mathrm{f}}^{\mathrm{ex}}) \tag{3}$$

where R_0 is the Förster radius, ν_{ex} the radiative exciton decay rate, $\Theta()$ the Heaviside step function and $E_{\mathrm{i}}^{\mathrm{ex}}$, $E_{\mathrm{f}}^{\mathrm{ex}}$ the exciton energies $E_{\mathrm{i/f}}^{\mathrm{ex}} = E_{\mathrm{i/f}}^{\mathrm{LUMO}} - E_{\mathrm{i/f}}^{\mathrm{HOMO}} - E_{\mathrm{b}}^{\mathrm{ex}}$ at the initial and final sites. Dexter-type exciton diffusion is implicitly accounted for as a double charge hopping process.

The waiting time before an event (hop or recombination) occurs is calculated as

$$\tau = -\frac{\ln(r)}{\Sigma_\nu} \tag{4}$$

where r is a random number drawn from a homogeneous distribution between 0 and 1 and Σ_ν is the sum of the rates of all possible events. The event that occurs after τ is selected randomly, using the rates of all possible events as weight factors. Energies, rates, and waiting time are recalculated after each event.

In order to simulate the transient response to a short light pulse, each simulation is started by creating an initial concentration c_0 of excitons on an otherwise empty calculation grid. Given the high photon energy employed in the experiments an equal excitation probability is assumed for all sites, in other words excitons are generated at fully random positions. Both in pulsed and steady-state simulations all charges are tracked in time, energy and position from the moment of generation till extraction or recombination.

Periodic boundary conditions in the x,y-directions were applied for both charge motion and (image and direct) Coulomb interactions; contacts laying in the z-plane are included unless stated otherwise and were implemented as perfect sinks. Independence of the results on the box size used in the calculations was assured.

As MC simulations are too time-consuming to allow true least-square fitting, estimates of the parameters were made entirely on basis of visual inspection of simulated data and kept unchanged during further simulations.

Most of the parameters in Supplementary Table 1, 2 were set upfront as determined in previous work[2,16,17] and kept fixed during all simulations. The lattice constant (= hopping distance) a_{NN} was fixed to a reasonable value that was previously extracted from curve fitting to SCLC data[6,15]. For PCDTBT:PC_{61}BM the lattice constant was further adjusted to better match the charge carrier recombination dynamics[2]. For TQ1:PC_{71}BM the ratio of the Förster radius and the lattice constant turned out to have no noticeable effect on the simulations and was set to a value of 2.2, which implies a Förster radius of 4 nm. Recombination rate for excitons was set to 1.9×10^{9} s^{-1} as determined from TRPL in Supplementary Fig. 3. The rate for CT complexes was set to 1×10^{8} s^{-1} to reproduce the experimental IQE≈0.85[13]. For PCDTBT:PC_{61}BM the Förster radius, the lattice constant and the recombination rates for excitons and CT complexes were adjusted to match the TRPL measurements (Supplementary Fig. 3) and experiments on charge carrier recombination dynamics[2]. See Supplementary Table 1, 2 for which experiments defined the simulation parameters.

As ν_0 is generally understood as related to some phonon frequency, it is not upfront clear why the different geometries of fullerene and polymer (but with somewhat similar carbon-carbon bonds) should give so different attempt frequencies. It should however be kept in mind that in the highly simplified Miller-Abrahams formalism (Eq. 1) also the overlap or transfer integral is contained in ν_0. It does not seem unlikely that the overlap integral of the rate limiting hops differs substantially between the spherical PCBM and the highly anisotropic polymer. Similar differences in ν_0 as obtained here were found from a detailed analysis of SCLC curves of pristine $PC_{61}BM$ and pristine MDMO-PPV[17].

Although our model is a crude approximation of ultrafast (and in literature suggested to be coherent) phenomena occurring on the fs-ps time scale, within the framework of our model the used parameters predict charge-transfer rates that are comparable to the experiment of Gélinas et al.[20]. As our lattice is 6-fold coordinated, within the framework of our model charge-transfer occurs approximately after a time 1/(6 x ν_0), where ν_0 is the attempt-to-hop frequency. Thus, following photo-generation, charge transfer occurs in ≈17 fs in TQ1:$PC_{71}BM$ and in ≈56 fs in PCDTBT:$PC_{61}BM$. These are reasonable values as compared to the measured ≈40 fs in PCDTBT:$PC_{61}BM$ (Gélinas et al.[20]). Also, detailed treatment of ultra-fast phenomena is not the main purpose of our model. The purpose is to use a *single* framework to describe a large number of experiments over a wide range of times, and to accomplish this by the least number of parameters – most of which are in fact determined by experiments.

Finally, we point out a technical detail when comparing the simulation parameters listed in Supplementary Table 1, 2 to those from other theoretical models. Specifically, the disorder value σ and attempt-to-hop frequency ν_0 that are used to successfully fit the experimental data employing the above model will differ from the disorder value that might (or might not) fit the experimental data using non-adiabatic Marcus theory for charge or exciton dynamics.

Supplementary Note 2 | Comparison of PA and PB bands for measuring hole thermalization. We point out that the photo-induced bleach (PB) band is the more appropriate choice to monitor hole thermalization dynamics as compared to the photo-induced absorption (PA) band. We consider the PA band to be more problematic due to the likely overlap of several contributing species: singlet excitons, triplet excitons, polarons, possibly not only from the donor but also (though weaker) from the acceptor. In recent literature the relative contributions of these species are disentangled via multivariate curve resolution (MCR) methods. However, such methods cannot treat dynamic spectra: the main assumption of MCR analysis are static spectra in energy of each individual species, which is not suitable for the present purposes. Moreover, the PA band probes the absorption between excited states, the absorption of which may (or may not) change as the carrier population thermalizes, making it difficult to quantify the energy loss to thermalization. Via the PB signature we are predominantly monitoring the energetic position of the hole, making the PB band the appropriate choice in this case.

Supplementary Note 3 | Explanation of how the TRMC/TA mobility was estimated. Since both of the THz and TRMC techniques in fact monitor the time-resolved conductivity $\sigma(t) = en(t)\mu(t)$, for the correct determination of the time-dependent mobility $\mu(t)$ the charge carrier concentration dynamics $n(t)$ in the same time range must be known. This was accomplished by combination with the time-resolved photo-induced absorption (PIA) data[5], allowing for the time-dependent mobility $\mu(t)$ in the commonly accessible time range to be estimated. As PIA only follows the hole population dynamics such analysis was made possible by the following observation: for TQ1:PC_{71}BM a purely bimolecular charge carrier concentration decay is observed[5] – charge carriers recombine in pairs, thereby both hole and electron densities should be similar. This allows for the direct conversion of the time-resolved conductivity $\sigma(t)$ to the time-dependent mobility $\mu(t)$ by the use of the PIA experimental estimate of $n(t)$. Similar arguments were used in case of THz, see Supplementary Fig. 8.

Supplementary Note 4 | Explanation for the high number of hops. The high number of hops during the diffusion-dominated loss step is because most of the hops occur downward in energy. This is confirmed by inspecting the minimum time it would take for an average charge carrier to complete such a number of hops, as can be done by the use of simulation parameters in Supplementary Table 1, 2. For example in case of electrons in TQ1:PC_{71}BM the number of hops (≈7000) times the inverse of the attempt to hop frequency ($1/\nu_0$, an estimate of the minimum time it takes between consecutive hops) results in $7000 \times 1/\nu_0$ ≈0.7 ns. This is in excellent agreement with the transition from diffusion- to drift-dominated motion as can be read from the thickened simulation traces in Figure 2 of the main text. The same analysis leads to 0.36 µs and 80 ns for the holes in TQ1:PC_{71}BM and PCDTBT:PC_{61}BM respectively, again in good agreement with Figure 2 of the main text. Thus, during the diffusion-dominated loss step, an average charge carrier mostly hops downward in energy.

Supplementary References

1. Etzold, F. *et al.* Ultrafast Exciton Dissociation Followed by Nongeminate Charge Recombination in PCDTBT:PCBM Photovoltaic Blends. *J. Am. Chem. Soc.* **133,** 9469–9479 (2011).
2. Howard, I. A., Etzold, F., Laquai, F. & Kemerink, M. Nonequilibrium Charge Dynamics in Organic Solar Cells. *Adv. Energy Mater.* **4**, 1301743 (2014).
3. Baranovskii, S. D. Theoretical description of charge transport in disordered organic semiconductors. *Phys. Status Solidi B* **251,** 487–525 (2014).
4. Murthy, D. H. K. *et al.* Origin of Reduced Bimolecular Recombination in Blends of Conjugated Polymers and Fullerenes. *Adv. Funct. Mater.* **23,** 4262–4268 (2013).
5. Andersson, L. M. *et al.* Unified Study of Recombination in Polymer:Fullerene Solar Cells Using Transient Absorption and Charge-Extraction Measurements. *J. Phys. Chem. Lett.* **4,** 2069–2072 (2013).
6. Pasveer, W. F. *et al.* Unified Description of Charge-Carrier Mobilities in Disordered Semiconducting Polymers. *Phys. Rev. Lett.* **94,** 206601 (2005).
7. Savenije, T. J., Ferguson, A. J., Kopidakis, N. & Rumbles, G. Revealing the Dynamics of Charge Carriers in Polymer:Fullerene Blends Using Photoinduced Time-Resolved Microwave Conductivity. *J. Phys. Chem. C* **117,** 24085–24103 (2013).
8. Němec, H., Kužel, P. & Sundström, V. Charge transport in nanostructured materials for solar energy conversion studied by time-resolved terahertz spectroscopy. *J. Photochem. Photobiol. Chem.* **215,** 123–139 (2010).
9. Hilt, O. & Siebbeles, L. D. A. Time and frequency dependent charge carrier mobility on one-dimensional chains with energetic disorder for polaron and Miller–Abrahams type hopping. *Chem. Phys.* **229,** 257–263 (1998).

10. Vukmirović, N., Ponseca, C. S., Němec, H., Yartsev, A. & Sundström, V. Insights into the Charge Carrier Terahertz Mobility in Polyfluorenes from Large-Scale Atomistic Simulations and Time-Resolved Terahertz Spectroscopy. *J. Phys. Chem. C* **116,** 19665–19672 (2012).

11. Pasveer, W. F., Bobbert, P. A. & Michels, M. A. J. Universality of AC conductivity: Random site-energy model with Fermi statistics. *Phys. Rev. B* **74,** 165209 (2006).

12. Ponseca, C. S. *et al.* Ultrafast Terahertz Photoconductivity of Bulk Heterojunction Materials Reveals High Carrier Mobility up to Nanosecond Time Scale. *J. Am. Chem. Soc.* **134,** 11836–11839 (2012).

13. Tang, Z. *et al.* Semi-Transparent Tandem Organic Solar Cells with 90% Internal Quantum Efficiency. *Adv. Energy Mater.* **2,** 1467–1476 (2012).

14. Kim, Y., Yeom, H. R., Kim, J. Y. & Yang, C. High-efficiency polymer solar cells with a cost-effective quinoxaline polymer through nanoscale morphology control induced by practical processing additives. *Energy Environ. Sci.* **6,** 1909–1916 (2013).

15. Bouhassoune, M., Mensfoort, S. L. M. van, Bobbert, P. A. & Coehoorn, R. Carrier-density and field-dependent charge-carrier mobility in organic semiconductors with correlated Gaussian disorder. *Org. Electron.* **10,** 437–445 (2009).

16. Melianas, A. *et al.* Dispersion-Dominated Photocurrent in Polymer:Fullerene Solar Cells. *Adv. Funct. Mater.* **24,** 4507–4514 (2014).

17. van Eersel, H., Janssen, R. A. J. & Kemerink, M. Mechanism for Efficient Photoinduced Charge Separation at Disordered Organic Heterointerfaces. *Adv. Funct. Mater.* **22,** 2700–2708 (2012).

18. Wang, E. *et al.* An Easily Synthesized Blue Polymer for High-Performance Polymer Solar Cells. *Adv. Mater.* **22,** 5240–5244 (2010).

19. Chu, T.-Y. *et al.* Highly efficient polycarbazole-based organic photovoltaic devices. *Appl. Phys. Lett.* **95,** 063304 (2009).

20. Gélinas, S. *et al.* Ultrafast Long-Range Charge Separation in Organic Semiconductor Photovoltaic Diodes. *Science* **343,** 512–516 (2014).
